# Spontaneous Heterotopic Pregnancy, Simultaneous Ovarian, and Intrauterine: A Case Report

**DOI:** 10.1155/2012/509694

**Published:** 2012-09-10

**Authors:** Francesca Basile, Cristina Di Cesare, Lorena Quagliozzi, Laura Donati, Marina Bracaglia, Alessandro Caruso, Giancarlo Paradisi

**Affiliations:** Department of Obstetrics and Gynecology, Catholic University of Sacred Heart, Largo A. Gemelli 8, 00168 Rome, Italy

## Abstract

Spontaneous heterotopic pregnancy is a rare clinical condition in which intrauterine and extrauterine pregnancies occur at the same time. The occurrence of an ovarian heterotopic pregnancy is a singular event as it comprises only 2.3% of all heterotopic pregnancies, extremely rare among women who conceive naturally. A case of a 28-year old patient was treated for spontaneously conceived heterotopic pregnancy. The patient was admitted to our center with lower abdominal pain and amenorrhoea. A transvaginal ultrasound scan showed an ovarian and an intrauterine heterotopic pregnancy. This was managed laparoscopically. Considering spontaneous pregnancies, every physician treating women of reproductive age should be aware of the possibility of heterotopic pregnancy. It can occur in the absence of any predisposing risk factors; only with an early diagnosis and treatment the intrauterine pregnancies will reach viability with a great chance of a favorable obstetric outcome.

## 1. Introduction

Heterotopic pregnancy (HP) is defined as the simultaneous presence of intrauterine (IUP) and ectopic pregnancy (EP). Commonly the EP is within the fallopian tube and uncommonly in the cervix or ovary [1]. Ovarian EPs by themselves are uncommon, accounting for only 1–3% of all ectopic pregnancies [2]. The estimated incidence of HP is between 1/8000 and 1/30,000 [3]. The occurrence of an ovarian HP is a singular event as it comprises only 2.3% of all HP [4]. However in the last decades there has been a significant increase of ectopic pregnancy and a subsequent increase of HP. This raised frequency has been attributed to several factors including higher incidence of pelvic inflammatory disease (PID) and the extended use of assisted reproductive technologies (ARTs). For patients who have been treated with ovulation-induction medication an incidence of 33/10,000 has been reported [5], while it is extremely rare among women who conceive naturally. Due to difficult preoperative diagnosis, it can be dangerous for mother and the simultaneous IUP. Commonly the management of HP is surgery, consisting on the removal of the EP. There have been many reports of using laparoscopy for the management. In this report we present a case with a spontaneous ovarian HP; seven similar cases previously reported in the literature have been reviewed. 

## 2. Case Report

A 28-year-old African (3 gravida, 0 para, 1 abortion) woman was admitted to the emergency room at the A. Gemelli University Hospital, Rome, on June 28, 2011, complaining lower abdominal-pelvic pain and generally feeling unwell. She reported vaginal bleeding the previous day. At this time, the patient was pregnant at 7th week of gestation and her last menstrual period was on May 16, 2011. She conceived spontaneously with no previously fertility treatment; she did no use any contraception in the interim; she had no history of pelvic inflammatory disease. Six years earlier she had a cesarean section delivery for a twin pregnancy and four years earlier a revision of uterine cavity for a miscarriage.

**Figure 1:**
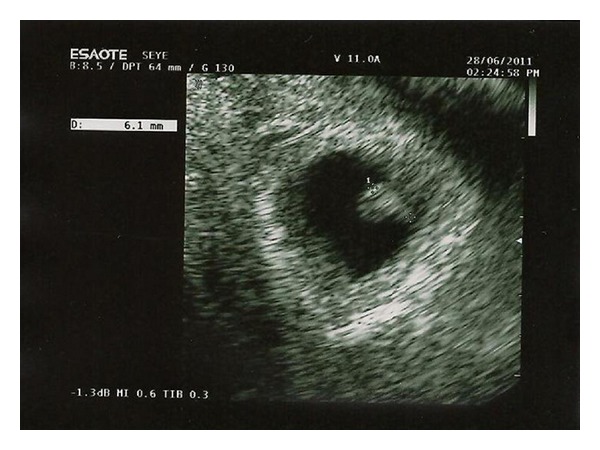
Transvaginal ultrasound of uterus showing a gestational sac of approximately 7 weeks.

**Figure 2:**
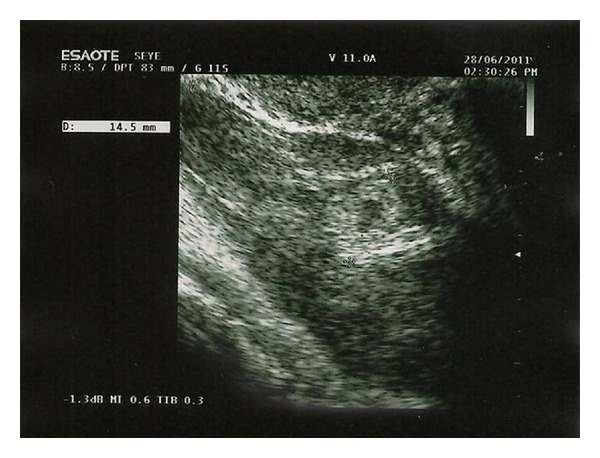
Transvaginal ultrasound of uterus showing left ovaric mass containing ovular material.

Abdominal examination revealed mild tenderness at the left lower abdomen. Pelvic examination revealed no bleeding from the uterine cervix. Uterus was enlarged with a tender palpable mass of diameter of 3-4 cm at the left annex, Pouch of Douglas was painful on palpation. Her hemoglobin concentration was 12.0 g/dL, the hematocrit was 36.4%, and white blood cell counts were 9,930/mm^3^. Her serum *β*-HCG was 55201 mU/mL. A transvaginal sonography showed the intrauterine gestational sac according to 7 weeks of pregnancy, a yolk sac of 4 mm and crown-rump-length of 6 mm with current cardiac activity (Figure 1). The right ovary was normal; in addition, there was a left ovaric mass suggestive of an ectopic pregnancy, which had total size of 8 cm (Figure 2). There was no heart beat in the ovaric pregnancy. The amount of free fluid in the pelvis was of 95 × 50 × 27 mm.

## 3. Operative Procedure

The woman was counseled and consented for an operative laparoscopy few hours later. A total of 900 cc of hemoperitoneum was aspirated. The uterus was enlarged according to the gestational age. The right annex was normal, and in the ovary was located a corpus luteum; the left annex presents a normal structure and morphology of the tube, except for a cystic formation of 1 cm diameter. The left ovary presented in the lower pole a circular bleeding formation of 3 cm diameter, probably of trofoblastic nature, that was partially removed and sent for extemporaneous histologic examination. In the upper pole was located a second corpus luteum. After the identification of ovular material in the removed tissue, the ovaric gestational sac was enucleated by the remaining healthy tissue. Duration of surgery was 60 minutes. Estimated intraoperative blood loss was 100 mL. Paracetamol was used for postoperative pain. The material was sent for definitive histologic examination and was confirmed chorionic villi suggestive of an ovarian HP. The postoperative course was uneventful. During the followup on the second postoperative day, the ultrasound was repeated and a viable intrauterine pregnancy was seen, confirmed by the fetal cardiac activity. The patient was discharged on the 6th postoperative day. Actually the viable pregnancy, at 3rd trimester, is followed in our Obstetric Department.

## 4. Discussion

The coexistence of intrauterine and extrauterine pregnancy, also known as heterotopic pregnancy, can occur in different forms: intrauterine pregnancy and tubal, abdominal, corneal, cervical or ovarian pregnancy. A previous review showed that most of extrauterine pregnancies were located in the fallopian tube (72.5%) [6]. An ovarian HP is a rare diagnosis with few reported cases. We reviewed the literature and only “5” cases of ovarian HP were spontaneous [7], while most of them were following clomiphene or ART use [8] (Table 1); HP may be considered, in fact, a consequence of modern reproductive medicine. Whereas the frequency of spontaneous HP varies from 1 : 10,000 to 1 : 50,000, the wide-spread use of ART may increase its incidence at nearly 1% in some series. Spontaneous HP is a potential fatal condition. Clinicians should maintain a high index of suspicion in all patients presenting with amenorrhea, abdominal pain, adnexal mass, peritoneal irritation, and enlarged uterus, even if an IUP has been confirmed. Suspicion should be higher in women with risk factors for an EP and in low risk women who have free fluid with or without an adnexal mass with an IUP. An HP is difficult to assess as pain and bleeding might be attributed to a threatened abortion. If ART is not involved, the index of suspicion of an HP is usually very low. This fact could cause more serious complications, such as a delayed diagnosis. The presence of an IUP, either viable or not, may actually mask the ectopic component of an HP, resulting in delay of diagnosis. A simultaneous IUP causes difficulties in the interpretation of ultrasound images. The ultrasound visualization of heart activity in both intrauterine and extrauterine gestations is important for diagnosis, but rare. During an ultrasound examination an ovaric pregnancy is easily misdiagnosed with a corpus luteum. The early diagnosis of HP is difficult; *β*-hCG alone is not helpful to diagnosis HP. The intrauterine pregnancy masks any underlying *β*-hCG changes from the extrauterine pregnancy and vice versa. Often the diagnosis is made during operation or after the histopathological report. Our case fulfills the Spiegelberg [9] four criteria for ovarian pregnancy: (1) the Fallopian tube with its fimbria should be intact and separate from the ovary; (2) the gestational sac should occupy the normal position of the ovary; (3) the gestational sac should be connected to the uterus by the ovarian ligament; (4) ovarian tissue must be present in the specimen attached to the gestational sac. In our case although the patient had no risk factors for EP, as previous PID or clomiphene use, an accurate evaluation of clinical signs associated with a specific ultrasound examination allowed a very early diagnosis.

The management of HP remains controversial. After diagnosis, the ectopic component is usually treated surgically, whereas the intrauterine component is expected to develop normally. An alternative treatment is the use of methotrexate, but it would be contraindicated in the presence of a viable intrauterine pregnancy. The traditional method of treating an ovarian pregnancy is laparoscopic wedge resection or ipsilateral oophorectomy. The advantages of laparoscopy over laparotomy in postsurgical recovery are well known. The gravid uterus should be handled carefully during the procedure. Early diagnosis and laparoscopic treatment provide good outcome without postsurgical inconvenience of laparotomy, with the advantage over medical treatment of an immediate result. Furthermore laparoscopic management avoids the risk of uterine handling and drying from open exposure, which may cause uterine irritability and postoperative spontaneous abortion; it is a definitive solution for the treatment of the ectopic pregnancy and at the same time it is safe for the viability of the intrauterine gestation. In Table 1 we summarized clinicopathological features of our case comparing it to previous cases reported in literature. Briefly, the most interesting characteristic is that only our case was managed laparoscopically with ovarian preservation. Previous cases are very old and probably fifteen years ago laparoscopic management was not very widespread like now. 

Considering spontaneous pregnancies, every physician treating women of reproductive age should be aware of the possibility of HP. It can occur in the absence of any predisposing risk factors. A high index of suspicion followed by an early surgical laparoscopic intervention can minimize maternal morbidity and preserve the developing of IUP. With early diagnosis and treatment, 70% of the intrauterine pregnancies will reach viability [14].

## Figures and Tables

**Table 1:** Clinicopathologic characteristics of previously spontaneous OEP.

	Milnor and Bowles 1940 [10]	Rannels 1953 [11]	Mulla and Johns 1958 [12]	Eyton-Jones 1968 [13]	Kuhl et al. 1995 [7]	Current case 2011
Age	28	32	41	21	41	28

Symptoms and signs	Nausea, abdominal pain, vomiting, and dyspnea	Irregular vaginal bleeding and severe low abdominal pain	Lower abdominal pain	Continuous pain in the epigastric and umbilical regions, nausea; vaginal bleeding	Abdominal pain, tenderness, loss of weight and vomiting	Lower abdominal-pelvic pain, mild tenderness at the left lower abdomen, vaginal bleeding

USG followup	/	/	/	/	IUP intact; a large mass originating from the left ovary, small amounts of free fluid were also seen	IUP intact, a left ovaric mass suggestive of EP, free fluid in the pelvis

Implantation site	Right ovary	Left ovary	Right ovary	Right ovary	Left ovary	Left ovary

Surgery	Laparotomic surgery: right salpingo-oophorectomy	Laparotomic surgery: left salpingo-oophorectomy	Laparotomic surgery: right salpingo-oophorectomy	Laparotomic surgery: removal of the right tube and ovary	Laparotomic surgery: removal of a large hematoma from the left ovary to the sigmoid colon	Laparoscopic surgery: ovaric gestational sac was enucleated with ovarian preservation

Pathology	Chorionic villi covered by decidua cells	The microscopic diagnosis: ectopic, ovarian pregnancy	Necrotic material of chorionic villi and a few viable decidual endometrial cells	Not specificated	Blood clot surrounded by placental (trophoblast) tissue originating from ovarian stroma	Chorionic villi with ovarian tissue
